# A Six-Year Examination of the Influence of Surgical Technique and Intraoperative Intraglandular Clostridium Botulinum Toxin Application in Salivary Gland Tumor Operations

**DOI:** 10.3390/jcm13226902

**Published:** 2024-11-16

**Authors:** Felix Johnson, Nora-Maria Burian, Matthias Santer, Verena Strasser, Teresa Steinbichler, Benedikt Hofauer, Anna Stenzl, Johanna Klarer, Robin Lochbaum, Haochen Lei, Hongyuan Cao, Gabriel Hillebrand, Amir Bolooki

**Affiliations:** 1 Department of Otorhinolaryngology, Medical University of Innsbruck, Anichstraße 35, A-6020 Innsbruck, Austria; 2 Department of Otorhinolaryngology, Head and Neck Surgery, Ulm University Medical Center, Frauensteige 12, 89075 Ulm, Germany; 3 Department of Statistics, Florida State University, 117 N. Woodward Ave., Tallahassee, FL 32306-4330, USA; 4 Department of Otorhinolaryngology, Klinikum Rechts der Isar, TUM School of Medicine and Health, Ismaninger Str. 22, 81675 Munich, Germany

**Keywords:** parotidectomy, extracapsular dissection, lateral parotidectomy, partial parotidectomy, clostridium botulinum toxin, intraoperative, intraglandular, pleomorphic adenoma, Warthin tumor, salivary gland tumor

## Abstract

**Introduction:** Salivary gland tumor operations are associated with complications including facial nerve dysfunction (FND) and salivary fistula. The objective of this study was to investigate the effect of extracapsular dissection (ECD) and the application of Clostridium botulinum toxin (CBT) in contrast to partial and lateral parotidectomy on complications. **Methods:** All salivary gland tumor operations performed within the last 6 years were retrospectively examined. Data were collected from electronic patient files from our otorhinolaryngology clinic. Total parotidectomies and submandibulectomies were not included in the analysis of CBT application. **Results:** In total, 418 cases were examined, including 84 (20%) malignant tumors. In total, 18 patients underwent ECD, 93 partial parotidectomy, 199 lateral parotidectomy, 76 total parotidectomy, and 32 submandibulectomy. The most common complication was transient FND (49%; *n* = 205; data available for 415 patients), which was measured at four days. Additional complications included salivary fistula (*n* = 56), infection (*n* = 49), bleeding or hematoma (*n* = 21). Preoperative facial nerve paralysis (*p* < 0.0001), pain (*p* < 0.0001), and a history of squamous cell skin carcinoma (SCC) (*p* < 0.001) were predictive of malignancy. The application of CBT did not reduce the risk of salivary fistula (*p*-value: 0.0182) and was associated with a higher combined complication rate (*p*-value: 0.0199). ECD was not associated with a lower likelihood for FND (*p* = 0.350). **Conclusions:** Preoperative pain, facial paralysis, or a history of SCC are predictors of malignancy. Use of CBT was not associated with a reduced risk of salivary fistula, but rather a higher complication rate.

## 1. Introduction

Salivary gland tumors account for 3–10% of all head and neck tumors [1] and represent a fair proportion of soft tissue surgery for head and neck surgeons. Removal of salivary gland tumors requires extensive surgical experience and complications are considered highly dependent on the surgeon’s expertise [2]. Complications following salivary gland tumor operations include wound infections, bleeding and hematoma, chronic pain, and Frey’s syndrome. Further complications include facial nerve dysfunction (FND) and salivary fistula. The surgical removal of tumors of the parotid gland is performed either via extracapsular dissection (ECD), partial parotidectomy, lateral parotidectomy (also known as superficial parotidectomy), via total parotidectomy or radical parotidectomy. Each approach has potential advantages and may be successful when appropriately performed.

A salivary fistula is a postoperative complication that may develop after a salivary gland operation. It typically presents when only part of the salivary gland has been resected. In such cases, the remaining gland tissue at the border of the resected surface continues to produce saliva. When the excreted saliva does not follow the normal anatomical drainage pathway, it may collect in the wound underneath the skin in what is known as a sialocele. In some cases, a salivary fistula may develop when the saliva drains through the sutured wound out of the skin. Frey’s syndrome, also known as gustatory sweating, typically occurs after parotid surgery due to aberrant postoperative neuronal regeneration [3]. Symptomatic Frey’s syndrome may be reported from patients and is confirmed through the Minor’s starch–iodine test [4].

Of further interest is to determine which perioperative risk factors may be associated with sialocele or salivary fistulas, which have an estimated incidence of 9.1% [5]. The type of operation and tumor entity may influence the likelihood of developing a fistula. The intraoperative application of intraglandular Clostridium botulinum toxin may play a role in changing the chance of complications. Clostridium botulinum toxin is typically applied postoperatively in severe cases of persistent salivary fistula or sialocele. Its effect typically gradually begins about 7–10 days after application [6,7].

Currently, there are three leading types of botulinum toxin A that are produced: onabotulinum toxin A (ONA; Botox^®^), incobotulinum toxin A (INCO; Xeomin^®^), and abobotulinum toxin A (ABO; Dysport^®^). ONA and INCO are considered to have similar dosing units, whereas ABO is considered to have a dosing equivalent 2–3 times that of ONA or INCO [8].

Some surgeons apply approximately 200 units of Clostridium botulinum toxin type A (ABO) in order to lower the risk of postoperative fistulas, while other surgeons do not believe that it plays a role in reducing complications. To the best of our knowledge, only one clinical study has been conducted evaluating the postoperative risk of sialocele or salivary fistula when Clostridium botulinum toxin is intraoperatively applied [9]. Here, a lower rate of salivary fistulas was noted, although these results were not statistically significant.

In this study, we seek to determine if the intraoperative intraglandular application of Clostridium botulinum toxin, which some surgeons favor, is associated with a lowered risk of postoperative salivary fistula. Furthermore, the potential risk of other perioperative complications, such as bleeding, facial nerve dysfunction (FND), chronic pain, infections, hematoma, Frey’s syndrome, first-bite syndrome, and emergency reoperation, will be investigated.

These perioperative factors are also to be examined in the stratification of the type of operation performed. Additionally, this study aims to describe the heterogeneous types of benign and malignant tumors that develop in salivary gland tumors in Austria, as this has rarely been investigated.

## 2. Methods

The study protocol is in accordance with the Declaration of Helsinki. The Institutional Ethics Board of the University Clinic of Innsbruck reviewed and approved the protocol; it was also approved by the local ethics committee (date: April 2024; protocol code EK Nmr. 1124).

### 2.1. Data Collection

In this monocentric retrospective study, data collection began by thoroughly inspecting the electronic patient medical database of our electronic patient file system (KIS, Klinisches Informationssystem, Oracle Health, Redwood Shores, CA, USA) at the Department of Otolaryngology and Head and Neck Surgery at the Medical University Clinic of Innsbruck to find patients who had undergone surgery for salivary gland tumors between 1 January 2018 and 1 May 2024. This included patients who had tumor operations of the parotid, submandibular, sublingual, or minor salivary glands. Patients who underwent an operation because of sialolithiasis or chronic sialadenitis were not included. Based on these criteria, 418 patients were included in the study. Following partial parotidectomy, lateral parotidectomy, or ECP, our surgeons will mix approximately 150–200 units of the Clostridium botulinum toxin type A (ABO) (Dysport^®^, Ipsen, München, Germany) with 2 mL of saline solution. The decision to utilize botulinum toxin is at the discretion of the individual surgeon. The decision to administer botulinum toxin is based on the extent of the resection and the amount of residual parotid tissue remaining in situ, factors that may lead to complications such as a salivary fistula or Frey’s syndrome. In principle, botulinum toxin is administered in all operations where parotid tissue remains in situ. In rare cases, if the dissection of the parotid fascia, which divides the lateral from the medial part, is highly successful and the fascia remains intact, the surgeon may opt to forego the administration of botulinum toxin in our clinic. Subsequently, the CBT solution is directly injected under direct visualization into the parotid tissue that has not been removed. This is not performed in cases of total parotidectomy or submandibulectomies.

Patients who received a submandibulectomy or total parotidectomy do not typically receive CBT and therefore, were not included in the analysis of the effect of salivary fistula. However, data from these operations were still included so as to compare complication rates.

Subsequently, a review of the medical literature was conducted to identify potential factors that could affect perioperative complications. Patient records were accessed and relevant data points were identified and entered into an Excel spreadsheet. The location and histology of the primary tumor, history of squamous skin cancer or melanoma, history of smoking, age and gender were noted. Additionally, we examined the patient’s history to determine if this was the first operation of a salivary gland tumor or if other operations had taken place and due to what tumor entity, if a facial paralysis or pain were preoperatively or postoperatively present. Medical records during and on the last day of stay on the ward were reviewed to determine early and late complications. Cases of post-operative bleeding or infections were noted. The intraoperative intraglandular application of Clostridium botulinum toxin was noted via surgical reports. Postoperatively, most patients presented to our clinic about 12 days to 14 days after the operation in order to have the sutures removed, to discuss histology, and to check the status of the wound. Ambulatory doctors’ notes concerning wound status, facial nerve function, as well as the presence or absence of salivary fistula were consistently assessed at this point in our electronic patient database and recorded. In some cases, data points were not retrospectively able to be noted as they were not described in patient files. These cases were excluded for the specific analysis of said characteristics. In this study, patients’ medical records were retrospectively examined to determine if patients reported gustatory sweating, chronic pain, or first-bite syndrome after the operation. These data were typically reported in after-treatment of patients around the 6-month mark.

### 2.2. Statistical Analysis

The compiled and documented data points were entered into cells of the statistical software. Prism 5 (GraphPad, Version 9.2.0, San Diego, CA, USA) and Microsoft Office Excel 2016 (Microsoft, Redmond, WA, USA) were used for data analysis. Contingency analysis was performed using one-tailed Fischer’s exact test. A correlation was seen as significant if the *p*-value was smaller than 0.05. The predictive power of individual symptoms in relation to malignancy was also investigated. We used descriptive statistics to determine the frequency distributions (*n* and %) of baseline characteristics of the cohort. For continuous variables, average distributions with standard deviation (SD) or medians were utilized.

The logistic regression was performed to adjust for the potential covariates when we studied the relationship between occurrences of complications and some predictors, such as different types of operation, and the injection of CBT. The potential covariates included gender, age, diagnoses (benign or malignant tumors) and the occurrence of a re-operation. We adjusted different diagnoses by treating them as categorical variables in the logistic regression model. To decrease the number of covariates, we combined the rare diagnoses (frequency < 10) into the group “other benign” and “other malignancy”.

**Figure 1:**
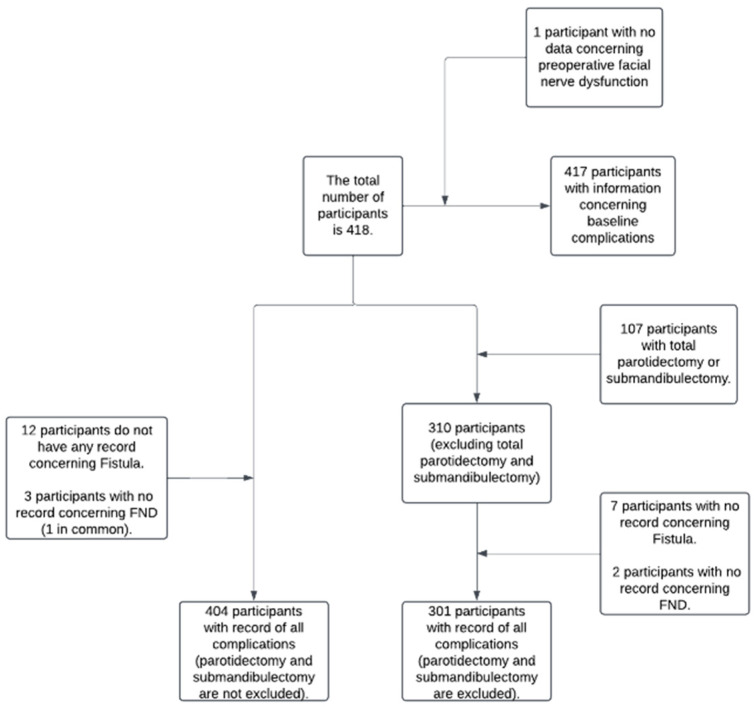
Flowchart illustrating the sample size and missing data points.

Missing data are presented in the flowchart in Figure 1.

## 3. Results

A total of 418 patients were included in the study (see Table 1). The most common tumor was the pleomorphic adenoma (PA; *n* = 167; 39.95%) including 10 recurrent PA (RPA), which were operated on a second time with a median of 12 years (std. dev. 6.37; mean 10.11) after the first operation. The second most common tumor was the Warthin tumor (WT; *n* = 125; 25.90%). The salivary gland most commonly affected was the parotid gland (*n* = 386; 92.3% of all tumors), followed by the submandibular gland (*n* = 32; 7.7% of all tumors; see Table 2). No tumors were removed from the sublingual gland. The most common malignant finding was lymph node metastasis due to squamous cell carcinoma of the skin (SCC; *n* = 31), followed by adenocarcinoma (*n* = 11).

For the entire cohort noted complication rates included infection (*n* = 49; 12%), hematoma/bleeding (*n* = 21; 5%) and need for reoperation (*n* = 11; 3%), Frey’s syndrome (*n* = 10; 2%), and chronic pain (*n* = 9; 2%). No patients were found to have first-bite syndrome.

Statistically significant correlations were observed between clinical features and malignity, which is shown in Table 3. These included preoperative facial paralysis (*p*-value: <0.0001), pain (*p*-value: <0.0001), or a history of squamous cell carcinoma (*p* < 0.001). No significant difference was noted postoperatively between the patients who had a malignant tumor vs. benign tumor and other complications (defined as bleeding, infection, Frey’s syndrome, chronic pain; *p*-value: 0.067).

**Figure 2:**
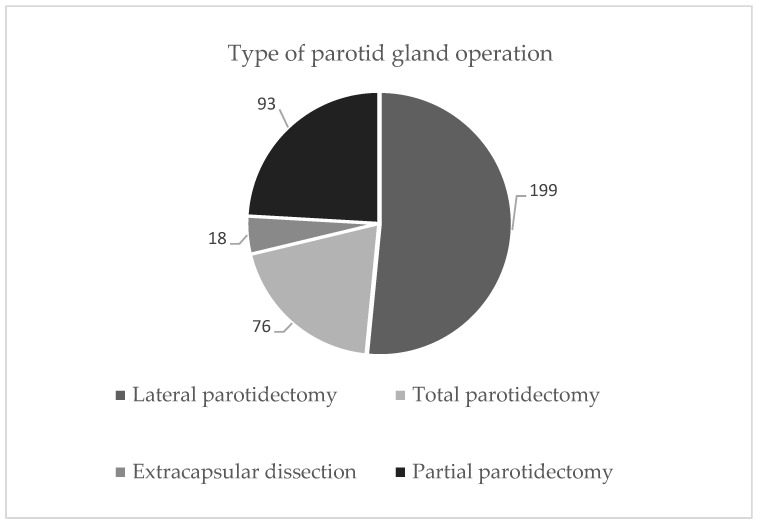
Type and frequency of operation performed in the parotid gland.

The most common complication was facial nerve dysfunction, which occurred in 49% of patients (*n* = 205; data available for 415 patients) measured at 4 days. At 12–14 days (data available for *n* = 202 patients) there was a recorded complete improvement in 26.8% (*n* = 54) of patients, partial improvement in 107 (53%), and no improvement in 20.3% (*n* = 41). The most commonly performed surgical technique was the lateral parotidectomy (*n* = 199; 48%), followed by the partial parotidectomy (*n* = 93; 22%) (see Figure 2). The lateral parotidectomy was defined as the complete removal of the superficial parotid gland with main facial nerve exposition. A partial parotidectomy was defined as the main facial nerve exposition with the partial removal of the superficial parotid gland. Extracapsular dissection was defined as the removal of a superficial portion of the parotid gland without exposition of the main facial nerve branch (*n* = 18; 4%). All total parotidectomies and submandibulectomies were excluded from the analysis of the complication rates for salivary fistula.

The logistic regression demonstrated that there was no association between the type of operation and complications (such as salivary fistula or facial nerve dysfunction, and other operation). The corresponding *p*-values are listed in Table 4. We also investigated the incidence of complications related to the administration of botulinum toxin and the type of surgery, excluding total parotidectomy and submandibulectomy (see Table 5 for details). As this study is retrospective, it was not possible to obtain all data. The missing data are included in parentheses.

A second operation was performed in 6% of cases. The rate of FND directly postoperatively in patients with one vs. two operations was 48.7% vs. 60.0% (*p* = 0.27). Other complications (defined as bleeding, infection, Frey’s syndrome, chronic pain) were more common after the second operation (18.6% vs. 26.9%), though this was not statistically significant (*p* < 0.2975).

Additionally, we found that the injection of CBT was associated with an increased rate of the salivary fistula (2.36; *p* = 0.024) and other complications (1.25; *p* = 0.030), but did not influence the rate of postoperative facial nerve dysfunction (0.33; *p* = 0.350) when the covariates and the type of operation are adjusted. The contingency table with total parotidectomy and submandibulectomy (illustrating these both excluded and included) can be found in Table 6 and Table 7.

## 4. Discussion

The cohort that received clostridium botulinum toxin consisted of 238 male and 180 female patients with a mean age of 58.29 years, who underwent a salivary gland tumor operation in Austria in the state of Tirol. Of these patients, a total of 274 patients received intraoperative intraglandular application of Clostridium botulinum toxin. While most studies examine clostridium botulinum toxin as an intervention for sialorrhea or salivary gland fistulas, the main focus of our study was to evaluate the possible prevention of such complications through intraoperative application. With 274 patients who received clostridium botulinum toxin in our cohort, our case numbers are significantly higher than in most other studies that investigated Clostridium botulinum toxin and its effect on sialorrhea or fistulas postoperatively [10,11,12]. Most of the literature with higher case numbers focuses mainly on neurological syndromes that lead to sialorrhea [13]. Studies focusing on primary salivary gland pathologies, such as sialadenitis or Frey’s syndrome, have reported lower-case numbers [11,14,15,16]. Furthermore, to improve the representativeness of our study no specific patient ethnicities were excluded. Since there are also multiple publications regarding clostridium botulinum toxin for neurological pediatric disease, compared to these studies, our cohort shows a much wider age range, raising the generalizability of our findings [13]. In this cohort, the most common type of tumor was the pleomorphic adenoma (PA) followed by the Warthin tumor (WT), which conforms to the demographic data presented in other studies.

Primary salivary gland cancers are a diverse group of tumors with more than 20 different histological subtypes. These tumors can vary greatly in their appearance in histopathology. However, there is often overlap between the different subtypes in terms of their histological, immunohistochemical, molecular, and biological features [17]. The most common primary malignant tumor was the adenocarcinoma (*n* = 11; 2.63% of all tumors). In most demographic studies, the most common primary malignancy is described as mucoepidermoid carcinoma [18]. However, in this study, only 4 cases were reported, as compared to 11 cases of adenocarcinoma. It is notable that, when considering all malignant carcinomas, the most common malignancy in this study was lymph node metastases due to squamous cell carcinoma of the skin (*n* = 31; 31%).

Squamous cell carcinoma of the skin is the most common type of cancer worldwide, with 70–80% [19] estimated to develop in the head and neck area, of which 5% are estimated to metastasize [19,20,21]. Other studies have reported the most malignant tumor of the salivary gland to also be metastasis due to squamous cell skin cancer [22]. This reinforces the need for regular and close check-ups including ultrasound examinations in patients with a history of SCC. Patients who preoperatively clinically presented with pain, FND, or a history of SCC or malignant melanoma were significantly predictive for a malignant tumor. As such, surgeons who operate on patients who preoperatively present with said symptoms should be preoperatively prepared for a malignant outcome.

Operative methods that have been described in preventing Frey’s syndrome include the use of local flaps that are interpositioned into the wound. However, this is typically primarily performed in order to improve cosmetic results or in advanced oncologic cases and is associated with further operative risks, and therefore rarely performed [23]. The use of postoperative radiotherapy in cases of refractory salivary fistula has been described [24]. However, its use is limited to its long-term adverse effects including xerostomia. The results of definitive studies concerning the association between surgical approaches and postoperative complications have been inconclusive. Some studies have indicated that ECD may be associated with a higher risk of postoperative facial nerve dysfunction (FND) and salivary fistula formation [25]. However, other studies have shown conflicting or differing results that advocate for this procedure [26,27].

In this study, the postoperative rate of FND was 49% measured at 4 days with a reported 26.8% having complete reconvalescence at 12–14 days. These results are similar to those described in other studies [28,29]. Although other studies have described lower rates of complications including FND after ECD [30], in our study, there was no association between the type of operation and FND (*p*-value: 0.3559) or salivary fistula (*p*-value: 0.6959). The rate of Frey’s syndrome in our study (2%) is comparable to those described in recent studies, which also described a 2% rate following ECD and in cases of lateral or superficial parotidectomy in 1.8% of cases [25]. However, studies have presented very different rates of Frey’s syndrome, some reporting symptoms in 23–43% of patients after 1 year [31]. In our study, the frequency of other complications (defined as bleeding, infection, Frey’s syndrome, and chronic pain; *p*-value: 0.6706) was also not significantly lower in patients with ECD as compared to partial and lateral parotidectomy. The total rate of bleeding requiring reoperation was 3%.

The extent of surgical gland excision is the subject of discussion in various studies. Many studies show higher rates of facial palsy when higher numbers of facial nerve branches were dissected intraoperatively. This mostly correlates with the extent of parotidectomy [32]. But there are still varying study results regarding other postoperative complications. While some studies describe varying fistula rates with no significant correlation to the extent of surgery others prove a correlation to residual gland parenchyma [33,34,35,36,37,38]. In summary, the surgical trend is towards minimally invasive resections in the form of extracapsular dissection (ECD). This is due to the fact that numerous studies have shown a reduced FND risk [26]. The use of Clostridium botulinum toxin in treating salivary fistulas postoperatively is common. However, its use prophylactically via direct intraoperative intraglandular application is rarely performed. Concerning the literature there seems to be only one other study examining intraoperative application of botulinum toxin. Lee Dong-Joo et al. [9] showed that the incidence of sialoceles was significantly lower in the cohort that received botulinum toxin. The rate of salivary fistula was, however, not shown to be significantly lowered. Our study showed that the use of Clostridium botulinum toxin was associated with a higher rate of salivary fistula (*p*-value: 0.0560). The main focus of the pre-existing literature is the use of botulinum toxin only after complications, such as fistulas, have occurred. Most studies generally describe injecting between 30–100 units of Clostridium botulinum toxin, though some studies have also applied up to 225 units [39].

Furthermore, the use and potential advantages of Clostridium botulinum toxin should also be evaluated concerning the number of patients needing treatment in order to prevent a potential salivary fistula. The number of units of toxin injected was approximately 200 units, with the cost of Clostridium botulinum toxin A (ABO) at our clinic amounting to approximately EUR 238 per 500 units. In this cohort, the intraoperative application of Clostridium botulinum toxin was associated with an increased risk of complications. Studies describe rates of salivary fistula approaching 9.5% [33], suggesting that, assuming the intraglandular intraoperative application of botulinum toxin is effective, in order to potentially prevent one salivary fistula, one would need to treat 10 patients. This may be economically unfeasible for many hospitals, as the costs to hypothetically treat one patient approach EUR 1190, assuming a 100% effectivity of the botulinum toxin. Moreover, in this study, the use of botulinum toxin was associated with a higher fistula rate.

Limitations of our study include its retrospective nature, which may be associated with problems in data acquisition, as some data may not have been documented. Besides the retrospective nature of this study, other limitations should be discussed. Information concerning whether the parotid fascia was reconstructed was not recorded. Some surgeons believe that reconstruction of the parotid fascia may lower the risk of salivary fistula or Frey’s syndrome [40]. Comorbidities associated with salivary gland tumor operations are difficult to quantify according to specific surgical techniques because postoperative rates of FND or salivary fistulas may be highly dependent on the type of tumor, location and size thereof, and the skill and expertise of the surgeon. Our study could be biased by the amount of surgical experience of the operating physician.

Regarding the high cost of botulinum toxin and the physician’s interest in acting as minimally invasive as possible, there can still be valuable information drawn from this study. The most sensible way to use clostridium botulinum toxin would be to only use it in high-risk cases. It is likely that clostridium botulinum toxin was administered in cases in which the surgeon deemed a high risk for complications. Such cases may include instances where resection margins and the salivary gland capsule were more difficult to identify due to inflammatory processes. This could result in more extensive damage to the gland capsule, thereby raising the risk of complications. Furthermore, the tumor’s location may have influenced the physician’s decision-making process, as studies have shown that tumors located in the middle of the gland are associated with higher rates of sialoceles and fistulas [5,38].

Few methods are available to prevent salivary fistula. One may potentially use anticholinergic medication in the postoperative setting to reduce the production of saliva. The transdermal application of scopolamine is usually first utilized as a treatment once salivary fistulas have already become clinically apparent. One study described the prophylactic perioperative application of transdermal scopolamine and noted a significant reduction in salivary fistula with a number needed to treat of 9.17 [41]. However, this method is rarely used prophylactically over the 2- to 4-week period, in which the risk of development of salivary fistula is at its highest. Additionally, such medications can lead to unwanted side effects such as urinary retention and confusion [42]. To effectively examine the use of Clostridium botulinum toxin, prospective randomized studies should be performed, and patients should be stratified according to groups defined by tumor type and surgical approach. This is important because there could be a potential source of bias regarding the use of CBT, namely the tendency for it to be employed in challenging settings in which surgery was already deemed to be a high-risk procedure.

## 5. Conclusions

In this monocentric retrospective cross-sectional study, we examined 418 patients who underwent salivary gland tumor operations. The high frequency of patients with lymph node metastasis due to SCC of the skin or malignant melanoma reinforces the need for active surveillance following head and neck skin cancer. The use of Clostridium botulinum toxin did not significantly reduce the rate of salivary fistula postoperatively but was associated with a higher rate of complications and salivary fistulas. A potential bias of this study is that Clostridium botulinum toxin may have been more likely to be utilized in difficult surgical cases where resection margins and the salivary gland capsule were more difficult to identify due to inflammatory processes. This could lead to more extensive surgical trauma raising complication risks. Additionally, a potentially high number of patients needing treatment may make this unproven prophylactic treatment of salivary fistulas economically unfeasible for many surgical departments.

## Figures and Tables

**Table 1:** Tumor histology and frequency.

Tumor Histology	Frequency	Percentage (%) of All Tumors
Pleomorphic adenoma	167	39.95
Warthin tumor	125	29.90
Lymph node metastasis (SCC)	31	7.42
Lymph node (benign)	14	3.35
Adenocarcinoma	11	2.63
Lymph node metastasis (malignant melanoma)	11	2.62
Lipoma	8	1.91
Cyst	8	1.91
Oncocytoma	6	1.44
Acinic cell carcinoma	6	1.44
Lymphoma	5	1.20
Mucoepidermoid carcinoma	4	0.96
Adenocystic carcinoma	4	0.96
Lymph node metastasis (Merkel cell carcinoma)	3	0.72
Myoepithelioma	2	0.48
Basal cell carcinoma	2	0.48
Basal cell adenoma	2	0.48
Carcinoma ex pleomorphic adenoma	2	0.48
Lymph node metastasis of a sarcoma	2	0.48
Secretory carcinoma	1	0.24
Schwannoma	1	0.24
Hemangioma	1	0.24
Secretory carcinoma	1	0.24
Undifferentiated carcinoma	1	0.24

**Table 2:** Summary of affected salivary glands and frequency of malignancy.

	Tumor Location	All Benign Tumors	All Malignant Tumors	Primary Salivary Gland Malignancy	Lymph Node Metastasis Due to SCC
Gl. parotis	386 (92.3%)	312	74	27	31
Gl. submandibularis	32 (7.7%)	22	10	10	0
Total	418	334	84	37	31

**Table 3:** Frequency and relationship between type of tumors and existence of complications (pain, facial paralysis and history of SCC). The number enclosed in parentheses represents the number of missing values within the specified class.

	Preoperative Pain		Preoperative Facial Paralysis (1)		History of SCC	
	Pain	No Pain	Facial Paralysis	No Facial Paralysis	History of SCC	No History of SCC
Benign	5	329	3	331	5	329
Malignant	30	54	10	73	36	48
	*p* < 0.0001		*p* < 0.0001		*p* < 0.0001	

**Table 4:** The number and the corresponding log odds ratio of salivary fistula and other complications stratified according to type of operation (patients with total parotidectomy or submandibulectomy were excluded). The covariates and the injection of botulinum toxin are adjusted.

	Total	Fistula	FND	Other
LP (Baseline)	199	30	83	45
PP	93	11	39	15
(log odd ratio)	/	−0.21	−0.04	−0.32
(*p*-value)	/	*p* = 0.598	*p* = 0.881	*p* = 0.360
ECD	18	2	5	3
(log odds ratio)	/	0.25	−0.54	0.11
(*p*-value)	/	*p* = 0.767	*p* = 0.350	*p* = 0.878

**Table 5:** Rate of salivary fistula and other complications stratified according to application of botulinum toxin and type of operation (patients with total parotidectomy or submandibulectomy were excluded). The number enclosed in parentheses represents the number of missing values within the specified class.

	Operation Type	Total	Fistula	Other Complications	FND
Clostridium botulinum toxin	ECD	8	2	3	3
	LP	175	29 (5)	42	74 (2)
	PP	80	11 (2)	14	34
	All	263	42 (7)	59	111 (2)
No Clostridium botulinum toxin	ECD	10	0	0	2
	LP	24	1	3	9
	PP	13	0	1	5
	All	47	1	4	16

**Table 6:** Contingency table of injection of botulinum toxin and type of operation (patients with total parotidectomy or submandibulectomy were excluded). The number in parentheses represents the number of missing values in that class.

	Salivary Fistula (7)		Other Complications		FND (2)	
	Salivary Fistula	No Salivary Fistula	Complications	No Complications	FND	No FND
CBT	42	214	59	204	111	150
No CBT	1	46	4	43	16	31
*p*-value	*p* = 0.0099		*p* = 0.0289		*p* = 0.2766	

**Table 7:** Contingency table of injection of botulinum toxin and type of operation (patients with total parotidectomy or submandibulectomy were not excluded). The number enclosed in parentheses represents the number of missing values within the specified class.

	Salivary Fistula (12)		Other Complications		FND (3)	
	Salivary Fistula	No Salivary Fistula	Complications	No Complications	FND	No FND
CBT	43	223	62	212	120	152
No CBT	13	127	18	126	85	58
*p*-value	*p* = 0.0560		*p* = 0.0124		*p* = 0.003	

## Data Availability

The original contributions presented in the study are included in the article, further inquiries can be directed to the corresponding author.

## Abbreviations

FND	Focal nerve dysfunction
ECD	Extracapsular dissection
SD	standard deviations
OR	Odds ratios
CI	confidence intervals
PA	Pleomorphic adenoma
RPA	Recurrent pleomorphic adenoma
WHO	World Health Organization
WT	Warthin’s tumor
CBT	Clostridium botulinum toxin
ONA	Onabotulinum toxin A
INCO	Incobotulinum toxin A
ABO	Abobotulinum toxin A
SCC	Squamous cell carcinoma
